# Editorial: Rapid research in action: lessons from the field

**DOI:** 10.3389/fsoc.2023.1216834

**Published:** 2023-06-20

**Authors:** Noémie Deom, Sigrún Eyrúnardóttir Clark, Ginger A. Johnson, Cecilia Vindrola-Padros

**Affiliations:** ^1^Rapid Research Evaluation and Appraisal Lab (RREAL), University College London, London, United Kingdom; ^2^United Nations Children's Fund (UNICEF), Geneva, Switzerland

**Keywords:** rapid research, rapid methods, rapid evaluation, rapid appraisal, rapid ethnography, rapid ethnographic assessment, participatory research

As we continue to recover from the devastating effects of the COVID-19 pandemic, we reflect on the lessons we learned in using evidence-based recommendations for policy and programming to control the spread of an infectious disease. Over the past several years, many research teams around the world worked tirelessly to generate high quality evidence in record time (Vindrola-Padros et al., 2020). Rapid appraisals and rapid assessments were implemented widely as diagnostic tools or to provide a “snapshot” of complex situations (Johnson and Vindrola-Padros, 2017). New innovations and interventions were rapidly evaluated and adapted using formative approaches such as rapid feedback and rapid cycle evaluations (Vindrola-Padros et al., 2021). Decades of work in the field of rapid research and evaluation meant that we were ready, from a methodological point of view, to respond to the pressures of adapting research design and implementation to the pace of the “real world” (Vindrola-Padros, 2021).

In this Research Topic, we synthesize, criticize and pay tribute to the use of rapid methods across disciplines during the COVID-19 pandemic and for other health emergencies and settings (pre- and post-pandemic). The authors featured within this Research Topic explore important questions about the practicalities of implementing rapid studies, the challenges they faced, the contributions of rapid research and evaluation, and the lessons learned that can be helpful for other teams and the future development of this field. Articles draw from community based, health systems and research carried out in clinical settings that explore a wide range of health-related topics such as cancer research, mental health, female contraception, prenatal stress, infection prevention, drug use, and the delivery of care in the context of complex health emergencies.

## Guidance and frameworks for rapid decision-making, insights, team-building, and building trust

The COVID-19 pandemic led to innovative methodological insights as researchers negotiated the need to obtain valuable qualitative data under short timeframes. The Rapid Insights (RI) approach developed by Chandler et al. uses data from a wide variety of stakeholders to understand their immediate needs and allow quicker decision-making. Williams et al. developed a template of steps to evaluate telehealth services that serve to produce rapid insights and ultimately aid decision-makers. With CLIP-Q (Collaborative and Intensive Pragmatic Qualitative Research), Horwood et al. propose pragmatic strategies for effective collaborations between academia and healthcare systems, thus harmonizing the quality standards of academic research with the demands and pressures of emergent issues. With limited to no access to their field site, Burn et al. explore the benefits and challenges of open data collection methods. Bright reminds us that there was a shift to the online sphere *prior* to the COVID-19 pandemic and describes research processes increasingly moving further online to coordinate teams and collect and analyze data in transnational health research contexts. Both Eaves et al. and Williams et al.'s use of rapid methodologies shows the transition of research, medical treatment and consultation from face-to-face to online platforms during the pandemic, which may have widened the access to care gap for those who cannot access digital technology.

Several articles in this Research Topic address the pragmatic choices rapid research necessarily entails. For instance, multi-country studies may require considering whether to implement a team-based or solo researcher approach (Wanat et al.), whether or not to transcribe all data (Wanat et al.; Suchman et al.) and whether common research terms such as “academic collaborations” or “sharing” may be fraught with legacies of extractivist science (Bright). Several papers also explore the challenges and benefits of diversity within teams. For instance, while Machin et al. give practical advice on how academic teams can develop long-term relationships with people with personal experience of mental health issues and involvement in research (or “lived experience researchers”), Higham et al. describe the challenges and benefits of conducting research with team members with dual clinical roles. Eaves et al. discuss the inclusion of community stakeholders in online ethnographies while Suchman et al. explore the degree of autonomy of local teams regarding cross-national analysis needs. Other papers address the challenges of conducting rapid research amidst crises. Howells and Dancause explore the difficulties of being a local researcher after a disaster, as LeNoble et al. reflect on the research team's wellbeing while navigating the challenges of a pandemic.

Building trust with participants when the time allotted to data collection is limited also requires practical strategies. In the context of a transnational global health study, Walton et al. promote regular meetings and the inclusion of stakeholders' interests and values in the results. In their rapid ethnography, Rosteius et al. recommend that researchers thrive to build an emotional connection while using professional inexperience to access detailed information. Both articles emphasize the importance of transparency and openness about all stages of the rapid research process with key stakeholders and participants.

## Use of rapid research for greater inclusivity and to reduce (in)equitable South-North relationships

Articles in this special edition also address issues of inequality when conducting rapid research and how rapid methodologies can (and have) focused explicitly on increasing the participation of affected populations throughout the research cycle. Eguiluz et al. identify key inequalities between Global South and Global North in relation to analyzing data and disseminating findings from their research during the COVID-19 pandemic. Their research focuses on ensuring equitable and safe partnerships with locally-led research, with methods adapted to protect the safety of the researchers. Bright uses rapid ethnographic methods to identify gender, economic and language barriers for setting up, administering, and enrolling patients into international clinical trial research. For example, in South Africa, some participants were uncertain whether or not they could seek care due to insufficient support from employers, husbands and/or tribal leaders. Scott et al. discuss key considerations for conducting rapid research with marginalized communities that have had unequal access to resources and power. Their research focuses on rural communities from Southern New Mexico (USA) and Vanuatu which lacked infrastructure and had prior negative experiences with research and researchers.

Pieterse uses rapid research methods to highlight funding disparities between different woredas (districts) in the Somali Region of Ethiopia, where the Somali regional government had been given more control of health budgeting. However, with this shift in autonomy came limited support for the heads of local woredas on how to govern health budgeting. Gender imbalance in leadership roles was also apparent with all-male leads of health bureaus and health centers. Johnson et al.'s rapid research shows conflicting COVID-19 policies across the USA may have disproportionately impacted Southeastern states which had the lowest vaccination rates and highest death rates in the country. The authors suggest that historically marginalized populations (e.g., due to race, disabilities, and poverty) in these locations were disproportionality affected by the pandemic and that unclear and often contradicting COVID-19 policies from the federal government, executive state governments, and local governments may have amplified the lack of knowledge and distrust around the seriousness of the virus. Gorbea Díaz et al. similarly discuss how insights gained from their rapid research highlighted how inequitable distribution of aid (especially to lower income residents) in Puerto Rico following the 2017 hurricanes, amplified pre-existing inequalities between marginalized populations and those with privilege and power.

## Localizing transnational interventions and evaluations for time-sensitive contexts

Transnational and global health-oriented articles included in this issue also raise important discussions on the role of rapid research in informing health interventions and evaluations in time-sensitive contexts. The work by Pieterse demonstrates in the Somali Region of Ethiopia, rapid research can be useful in (re)orienting planned interventions to the practical realities of resource-constrained settings. Both Bright and Suchman et al., discuss how transnational research also requires flexible methodologies which can be adapted to local contexts as needed. Rapid research techniques (such as rapid ethnographic inquiries and qualitative analysis) were incorporated into their studies in order to meet multiple objectives of large-scale multi-sited studies while also paying attention to local needs and priorities. For example, Suchman et al. detail a concurrent combination of more traditional analysis and rapid qualitative analysis methodologies to accommodate linguistic differences and to meet multiple research objectives. In fact, a number of authors similarly address navigating the boundaries between long-term and short-term studies, or traditional vs. more rapid methods. For instance, Wanat et al. address the issue of what makes research rapid, while Jones et al. reflect on their experience adapting a longer-term study to rapid research in order to respond to an unfolding health emergency.

To analyze healthcare services in Australia during the COVID-19 pandemic, Williams et al. describe how rapid evaluation methods (REM) were tailored to their specific context and stakeholder environments. Using a case study of a rapid evaluation of telehealth in pediatric care, this article shares a step-by-step template for evaluations of telehealth services (including enablers and challenges) most useful for informed decision-making by government health providers, pediatricians and families.

## The future of rapid research

The themes identified in this introduction also point to areas for future development in this field. One important area of focus will need to be the quality of rapid research and evaluation (keeping in mind that reduced timeframes might lead to research that ends up being rushed instead of rapid). The Rapid Research Evaluation and Appraisal Lab (RREAL) is currently designing the first Standards for Rapid Evaluation and Appraisal Methods (STREAM), which seek to improve the transparency and completeness of reporting in rapid evaluations and appraisals (https://osf.io/nhfm3/).

The papers in this Research Topic also highlight important questions in relation to the scale of research, particularly in the case of qualitative research, which tends to rely on the use of small and rich datasets. An interesting area of future exploration in the field of rapid qualitative research and evaluation will be the development and use of larger datasets, crossing disciplinary boundaries and drawing from digital tools traditionally applied in the field of “Big Qual Data”. These tools can facilitate the rapid analysis of qualitative data to better enable the use of findings in near real-time to inform changes in policy and practice. RREAL is currently conducting research in this field, more information can be found here: https://osf.io/b85xs/.

Key questions are raised in this volume and elsewhere regarding how we can create meaningful relationships with patients, carers and members of the public so they can properly engage with the topics we are studying, how we are studying them, who is included in research and how findings are used. Patient and public involvement and engagement (PPIE) in rapid research has particular challenges that might not be present in longer studies (Gilchrist et al., 2022), yet important work is currently underway to develop a model for involvement and engagement that can be suitable for rapid timeframes. One example is SPRINT (Strategies for Patient and Public Involvement in Research in Time-Sensitive Contexts), a network of organizations working on PPIE that can operate under a rapid response model so the views, preferences and needs of patients and members of the public can remain at the center of rapid research and evaluation.

The future of rapid research is ripe for experimentation and new developments. The field of rapid research and evaluation has used its rich history of rapid ethnographic assessments, rapid appraisals, rapid ethnographies and rapid evaluations to mature into a distinct field of inquiry, with its own approaches, contributions and challenges. As we move on to new developments, we will need to face the challenges ahead for developing strategies to address the issues and key questions raised by the authors in this Research Topic—focusing on the quality of rapid research, the expansion of its scale (while still retaining localized knowledge and contexts), and the development of inclusive models of research and evaluation.

## Author contributions

ND, SC, GJ, and CV-P developed the editorial, managed the author submissions, and logistical requirements of this Research Topic. All authors contributed to the article and approved the submitted version.

## In memoriam

Dr. Dakhina Mitra. A passionate advocate for children's rights and participatory research. A generous sharer of knowledge who believed research can change the world. We do too.
